# Immediate high-dose intravenous immunoglobulins followed by direct thrombin-inhibitor treatment is crucial for survival in Sars-Covid-19-adenoviral vector vaccine-induced immune thrombotic thrombocytopenia VITT with cerebral sinus venous and portal vein thrombosis

**DOI:** 10.1007/s00415-021-10599-2

**Published:** 2021-05-22

**Authors:** Tilmann Graf, Thomas Thiele, Randolf Klingebiel, Andreas Greinacher, Wolf-Rüdiger Schäbitz, Isabell Greeve

**Affiliations:** 1 Department of Neurology, Evangelisches Klinikum Bethel, OWL University Hospital, Bielefeld University, Bielefeld-Bethel Campus, Bielefeld, Germany; 2 Department of Transfusion Medicine, Institute for Immunology and Transfusion Medicine, University Medicine Greifswald, Greifswald, Germany; 3 Department of Neuroradiology, Evangelisches Klinikum Bethel, OWL University Hospital, Bielefeld University, Bielefeld-Bethel Campus, Bielefeld, Germany

Dear Sirs,

Here, we report a case of a 29-year-old male public health care professional, vaccinated with the recombinant adenoviral vector encoding the spike protein antigen of SARS-CoV-2 (ChAdOx1 nCov-19, AstraZeneca) on the 29th of March (day 1). Nine days later, he developed headache and abdominal pain, on day 12 emesis and abdominal cramps. On day 14, he was urgently admitted to a hospital due to severe headache and hematemesis. Upon admission, thrombocytopenia of 32/nL was detected. Gastroscopy showed diffuse mucosal bleeding. On MR imaging of the brain, a complete thrombosis of the left transverse and sigmoid sinus down to the left proximal jugular vein was demonstrated (Fig. 1a). The patient was immediately transferred to the Department of Neurology in our hospital. He had no neurological deficits at that time point and the MR imaging showed no involvement of the parenchyma and no bleeding due to the congestion of the sinus veins. An abdominal CT angiography revealed extensive thrombosis of the mesenteric and portal vein, explaining the profuse bleeding of the stomach. The novel vaccine-induced immune thrombotic thrombocytopenia (VITT), first described in March 2021, was suspected in this otherwise healthy young man not been exposed to heparin before [1, 2]. To inactivate the suspected pathogenic platelet factor-4 (PF4)-heparin antibodies through Fc receptor blocking, treatment with high-dose immunoglobulins (IVIG) at a dose of 1 g/kg body weight on day 1 and day 2 was started [3, 4]. During the first 24 h, the platelet count raised only marginally, but after 48 h, platelet count improved to 98/nl (Fig. 2). The severe multilocular thrombosis was treated with non-heparin-based anticoagulation by the direct thrombin inhibitor argatroban beginning immediately after the first dose of IVIG [5]. Due to its short plasma half-life time and the potential to be applied continuously, argatroban is superior to other non-heparin-derived anticoagulants in this severe condition with imminent profuse bleeding [5]. The efficacy was controlled by frequent measurement of the partial thromboplastin time (PTT) that should be elevated up to 1.5 times from normal (50–60 s) (Fig. 2). During the first night in our hospital, after application of 90 g IVIG, with a platelet count of 40/nl and a PTT of 50 s, the patient suffered two subsequent epileptic seizures as a consequence of a new left temporo-parietal intracranial hemorrhage found on CCT scan (Fig. 1c). The patient developed moderate aphasia and apraxia and antiepileptic drugs were started. After the second course of 90 g IVIG and successive normalization of the platelet count (Fig. 2), decline of the D-Dimers from 65.7 mg/L at presentation to 12.32 mg/L after 48 h (Fig. 2) and continuous application of i.v. agatroban with a mean PTT time of 42 s (Fig. 2), the patient improved to a slight aphasic syndrome. The transverse sinus successively recanalized as demonstrated on day 16 of treatment (Fig. 1b), as did the portal and mesenteric veins. Lactate serum levels stayed normal and the patient had no further abdominal discomfort. Antibody-mediated PF4-dependent platelet activation was confirmed in a blood sample taken before IVIG treatment as described [1] several days after the patient had recovered.

**Fig. 1:**
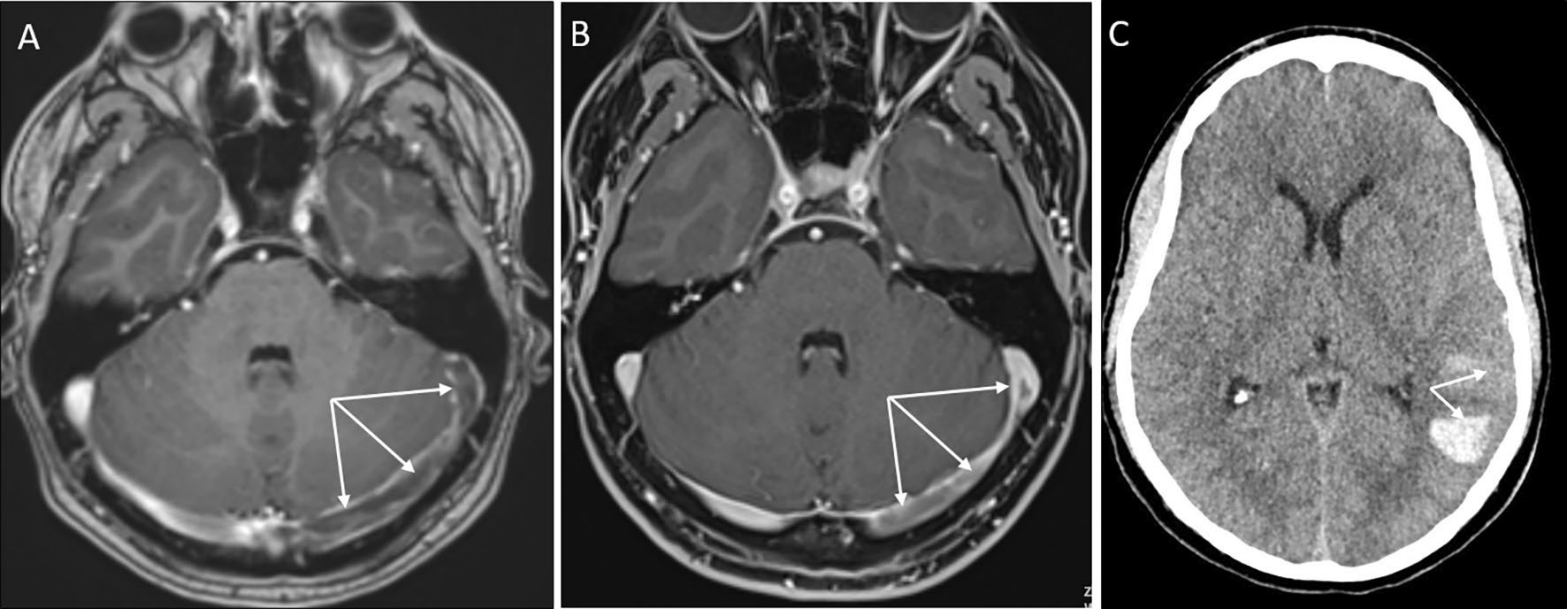
Imaging (MRI, CT) of the case report. **a**, **b** T1-weighted contrast-enhanced axial MR images on admission (**a**) and at 2-week follow-up (**b**). On admission, pronounced thrombosis is disclosed within the left transverse and sigmoid sinus, with progressive recanalization on follow-up MRI (arrows). **c** Plain head CT, displaying left-sided temporal hemorrhage

**Fig. 2:**
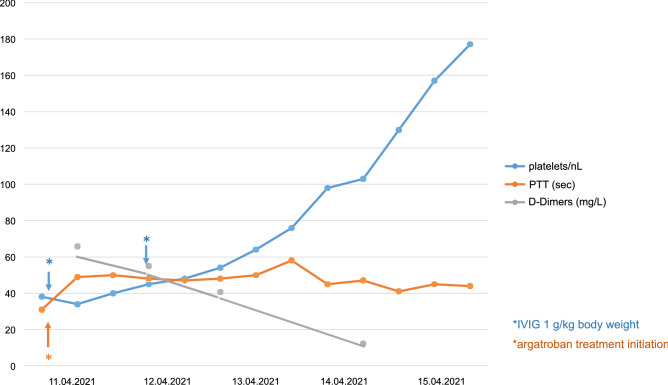
Platelet counts per nL, concentration of D-Dimers (mg/L) and partial thromboplastin time (PTT) in seconds are shown over time. Application of intravenous immunoglobulins (IVIG) and of the direct thrombin inhibitor argatroban is indicated by arrows

In conclusion, this case illustrates that immediate administration of high-dose IVIG and anticoagulation with direct thrombin inhibitor, avoiding application of platelet concentrates and initial administration of heparin, appears to be crucial in patients with life-threatening VITT even before the pathogenic PF4-heparin antibody is detected. Of the 16 published patients (11 in Germany and Austria, 5 in Norway) with unusual thrombotic events, 9 died of VITT [1, 2]. Only those patients who received high-dose IVIG close to the diagnosis of thrombocytopenia and thrombotic events with avoidance of platelet substitution further enhancing thrombus formation had a favorable prognosis in this otherwise devastating disease.

## References

[1] Greinacher A, Thiele T, Warkentin TE, Weisser K, Kyrle PA, Eichinger S (2021). Thrombotic thrombocytopenia after ChAdOx1 nCov-19 vaccination. N Engl J Med.

[2] Schultz NH, Sørvoll IH, Michelsen AE, Munthe LA, Lund-Johansen F, Ahlen MT, Wiedmann M, Aamodt AH, Skattør TH, Tjønnfjord GE, Holme PA (2021). Thrombosis and thrombocytopenia after ChAdOx1 nCoV-19 vaccination. N Engl J Med.

[3] Greinacher A, Selleng K, Warkentin TE (2017). Autoimmune heparin-induced thrombocytopenia. J Thromb Haemost.

[4] Warkentin TE (2019). High-dose intravenous immunoglobulin for the treatment and prevention of heparin-induced thrombocytopenia: a review. Expert Rev Hematol.

[5] Gray A, Wallis DE, Hursting MJ, Katz E, Lewis BE (2007). Argatroban therapy for heparin-induced thrombocytopenia in acutely ill patients. Clin Appl Thromb Hemost.

